# Assessment of thoracic aorta in different cardiac phases in patients with non-aorta diseases using cardiac CT

**DOI:** 10.1038/s41598-021-94677-5

**Published:** 2021-07-26

**Authors:** Xue Zheng, Yu-jiao Deng, Fu-Gang Han, Jin-Rong Zhou, Li Luo, Jing Chen

**Affiliations:** Department of Radiology, The Affiliated Hospital of Southwest Medical University, 25# Tai Ping Street, Luzhou, 646099 Sichuan China

**Keywords:** Computational biology and bioinformatics, Medical research

## Abstract

The aim was to evaluate the thoracic aorta in different cardiac phases to obtain the correct cardiac phase for measuring the maximum diameter required to predict aortic disease. Cardiac CT was performed on 97 patients for suspected coronary artery disease. The average diameter of ascending (AAD) and descending aorta (DAD) in the plane of pulmonary bifurcation, in the plane of the sinus junction (AAD [STJ] and DAD [STJ]), descending aorta in the plane of the diaphragm (DAD [Dia]), the diameter of the main pulmonary artery (MPAD), distance from the sternum to the spine (S-SD), and distance from the sternum to the ascending aorta (S-AAD) were assessed at 20 different time points in the cardiac cycle. Differences in aortic diameter in different cardiac phases and the correlation between aortic diameter and traditional risk factors were analyzed by the general linear mixed model. The diameter of the thoracic aorta reached the minimum at the phase of 95–0%, and reached the maximum at 30–35%. The maximum values of AAD, AAD (STJ), DAD, DAD (STJ), and DAD (Dia) were 32.51 ± 3.35 mm, 28.86 ± 3.01 mm, 23.46 ± 2.88 mm, 21.85 ± 2.58 mm, and 21.09 ± 2.66 mm, respectively. The maximum values of MPAD/AAD and DAD/AAD (STJ) were 0.8140 ± 0.1029, 0.7623 ± 0.0799, respectively. The diameter of the thoracic aorta varies with the cardiac phase. Analyzing the changes in aortic diameter, which can be done using cardiac CT, could provide a more accurate clinical measurement for predicting aortic disease.

## Introduction

The aorta is the largest elastic artery of the human body and is not only the conduit of blood transportation but also the storage organ for blood. Aortic diameter is an important predictor of cardiovascular disease, and the maximum diameter of the aorta, especially the ascending aorta, is used as a parameter to evaluate the risk and prognosis of aortic dissection and aneurysm rupture^1–5^. Therefore, accurate information on the size of blood vessels has a crucial impact on clinical decision-making, intervention, and operation decisions for aortic diseases^5,6^.

It is a well-established fact that aortic diameter changes during the cardiac cycle, and many studies have confirmed that aortic diameter differs in systole and diastole^7–11^. However, the aortic diameter is usually measured during a random phase of the cardiac cycle, and the result might be the largest or the smallest or in between, which leads to an inaccurate evaluation of aortic diameter. This can especially affect the choice of type and size of an aortic intraluminal graft. Multi-slice spiral computed tomography (CT) combined with retrospective electrocardiogram (ECG) gating could provide heart images with high time resolution and reconstruct multi-phase images of the whole cardiac cycle^12,13^. Therefore, it is recommended to use the technology of ECG gated CT acquisition for evaluating the diameter of blood vessels accurately (especially ascending aorta) instead of motion artifacts^14^.

In recent years, many scholars have studied how aortic diameter changes with the cardiac cycle^8,10,11,15^. However, most of these studies have only evaluated the change of diameter in systole and diastole phases and focused on the dynamic change of the aortic root but ignored the changes of the ascending and descending aortas. Moreover, different researchers have set different time points for systole and diastole in the cardiac cycle, which makes the assessment of the aortic diameter inconsistent in the cardiac phase and results in different dimensions for the same structure.

Therefore, the first aim of our study was to assess the dynamic changes of the diameter and relative ratios of the thoracic aorta in 20 cardiac phases. We used a more detailed division of cardiac cycles compared with other studies and information on accurate cardiac phases for the measurement of the maximum diameter, which is required to determine aortic disease. The other purpose of our study was to explore correlations with traditional cardiovascular risk factors.

## Results

### Baseline characteristics

We enrolled a total of 97 subjects (58 males, 39 females) with a mean age of 53.30 ± 10.68 years. The clinical characteristics of the study population are shown in Table 1. There were 31 subjects with hypertension, 37 subjects with hyperlipemia, and 15 subjects with hyperglycemia. The number of smokers and drinkers was 29 and 5, respectively.

**Table 1:** General clinical characteristics.

Variable	$\mathrm{N}(\%)/\overline{x }\pm s$
Age	53.30 ± 10.68
BMI	24.00 + 3.58
BSA	1.71 ± 0.19
**Gender**	
Female	39 (40.2)
Male	58 (59.8)
**Hypertension**	
N	66 (68.0)
Y	31 (32.0)
**Hyperlipemia**	
N	60 (61.9)
Y	37 (38.1)
**Hyperglycemia**	
N	82 (84.5)
Y	15 (15.5)
**Smoke**	
N	68 (70.1)
Y	29 (29.9)
**Drink**	
N	92 (94.8)
Y	5 (5.2)
**Inheritance**	
N	56 (57.7)
Y	41 (42.3)

### Aortic diameter characteristics

**Figure 1:**
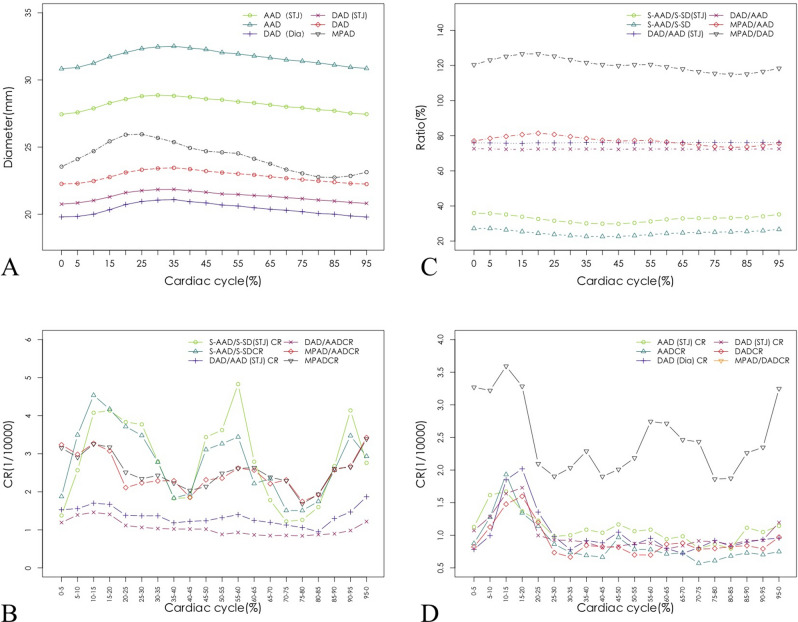
Relationship between aorta and cardiac cycle (with R (Version 3.6.1, R Core Team, 2019) software. URL: https://www.r-project.org/). (**A**) The relationship between aortic diameter and cardiac cycle, (**B**) the relationship between the change ratio of aortic diameter and cardiac cycle, (**C**) the relationship between aortic ratio and cardiac cycle, (**D**) the relationship between the change ratio of aortic ratio and cardiac cycle.

In different phases of the cardiac cycle, the differences of the aortic diameter were statistically significant (p < 0.05) (Fig. 1A; Table S1). The diameter of all arteries increased gradually from T = 0% when diameter was at minimum, and then increased slowly to the maximum when T = 35%, except DAD which was minimum at a cardiac phase of 95%, and AAD(STJ) which was maximum at a cardiac phase of 30%. The maximum values of AAD, AAD (STJ), DAD, DAD (STJ), and DAD (Dia) in the cardiac cycle were 32.51 ± 3.35 mm, 28.86 ± 3.01 mm, 23.46 ± 2.88 mm, 21.85 ± 2.58 mm, and 21.09 ± 2.66 mm, respectively, and the minimum values were 30.83 ± 3.85 mm, 27.44 ± 3.16 mm, 22.24 ± 2.88 mm, 20.75 ± 2.61 mm, and 19.80 ± 2.71 mm, respectively. And the peak phases were presented in histogram in Figure S1. The differences between the maximum and minimum AAD, AAD (STJ), DAD, DAD (STJ), and DAD (Dia) in a cardiac cycle were 7.00%, 7.45%, 6.94%, 8.02%, and 6.95%, respectively. The most dramatic change in the AAD in a cardiac cycle was during the 10% ~ 15% cardiac phase, while that of the DAD was during the 15% ~ 20% cardiac phase (Fig. 1B; Table S2).

Among the 20 different time points of the cardiac cycle, MPAD/AAD obtained the maximum value at a cardiac phase of 20% (MPAD/AAD = 0.8140 ± 0.1029) and the minimum value at 80% (MPAD/AAD = 0.7335 ± 0.0981); DAD/AAD (STJ) obtained the maximum value at a cardiac phase of 90% [DAD/AAD (STJ) = 0.7623 ± 0.0799] and the minimum value at 15% [DAD/AAD (STJ) = 0.7560 ± 0.0814] (Fig. 1B,C; Table S3). There was no statistical significance in the changes of DAD/AAD in the cardiac cycle (*p* = 0.982). And the rates of change in every 5% interval were shown in Fig. 1D and Table S4.

Using the general demographic characteristics and clinical characteristics as covariates and the arterial diameter or the ratio of arterial diameter as dependent variables, the generalized linear mixed effect model was established to obtain the correlation between traditional cardiovascular risk factors and the aorta (Tables 2, 3, Tables S5–S9). The correlation between aortic diameter in all planes and age in this study was statistically significant. For every 1-year increase in age, the mean diameter of AAD was higher by 0.156 mm, the mean diameter of DAD was higher by 0.130 mm, the mean diameter of DAD (Dia) was higher by 0.138 mm, and the mean diameter of DAD (STJ) and AAD (STJ) were both higher by 0.111 mm. The correlation between BSA (Body surface area) and aortic diameter at all levels except AAD in this study were statistically significant. For every 1.0 m^2^ increase in BSA, the mean diameter of DAD, DAD (STJ), DAD (Dia) and AAD (STJ) was higher by 7.743 mm, 5.994 mm, 6.515 mm, 4.022 mm, respectively. AAD (STJ), DAD (STJ) and DAD (Dia) was gender-related with the diameter in males was 1.548 mm, 1.120 mm, 1.232 mm wider than that in females, respectively.

**Table 2:** Single factor analysis (quantitative indicators).

Dependent variable	AAD	DAD	DAD (Dia)	DAD (STJ)	AAD (STJ)
**Covariant**					
Age					
B	0.165	0.092	0.100	0.077	0.081
*p*	** < 0.001**	** < 0.001**	** < 0.001**	**0.001**	**0.006**
Height					
B	− 0.461	12.627	11.936	11.887	9.561
*p*	0.916	** < 0.001**	** < 0.001**	** < 0.001**	**0.011**
Weight					
B	0.020	0.114	0.096	0.103	0.067
*p*	0.503	** < 0.001**	** < 0.001**	** < 0.001**	**0.009**
BSA					
B	0.741	7.076	6.296	6.480	4.442
*p*	0.695	** < 0.001**	** < 0.001**	** < 0.001**	**0.006**
BMI					
B	0.079	0.283	0.224	0.251	0.129
*p*	0.436	** < 0.001**	**0.003**	** < 0.001**	0.145

**Table 3:** Single factor analysis (qualitative indicators).

Dependent variable	AAD			DAD					
	$\overline{x }\pm s$	t	p	$\overline{x }\pm s$	t	p			
**Covariant**									
Gender									
Male	31.83 ± 3.92	0.585	0.560	23.69 ± 3.09	4.009	** < 0.001**			
Female	31.40 ± 3.04			21.49 ± 1.90					
Hypertension									
N	31.07 ± 3.73	2.436	**0.017**	22.23 ± 2.80	3.026	**0.003**			
Y	32.91 ± 2.93			24.04 ± 2.67					
Hyperlipemia									
N	31.47 ± 3.46	0.675	0.502	22.57 ± 2.64	1.046	0.298			
Y	31.97 ± 3.79			23.19 ± 3.20					
Hyperglycemia									
N	31.59 ± 3.78	0.484	0.629	22.87 ± 2.90	− 0.525	0.601			
Y	32.07 ± 2.31			22.45 ± 2.79					
Smoke									
N	31.67 ± 3.43	− 0.029	0.977	22.47 ± 2.60	1.795	0.076			
Y	31.64 ± 3.96			23.60 ± 3.33					
Drink									
N	31.58 ± 3.63	0.943	0.348	22.78 ± 2.92	0.433	0.666			
Y	33.12 ± 2.57			23.35 ± 2.09					
Inheritance									
N	31.85 ± 3.75	− 0.604	0.547	22.47 ± 2.92	1.366	0.175			
Y	31.41 ± 3.36			23.27 ± 2.78					

Dependent variable	DAD (Dia)			DAD (STJ)			AAD (STJ)		
	$\overline{x }\pm s$	t	p	$\overline{x }\pm s$	t	p	$\overline{x }\pm s$	t	p
**Covariant**									
Gender									
Male	21.36 ± 2.84	4.763	** < 0.001**	22.22 ± 2.67	4.726	** < 0.001**	29.04 ± 3.37	3.596	** < 0.001**
Female	18.97 ± 1.71			19.96 ± 1.68			26.86 ± 2.22		
Hypertension									
N	19.89 ± 2.58	2.812	**0.006**	20.82 ± 2.41	2.863	**0.005**	27.77 ± 2.97	1.839	0.069
Y	21.48 ± 2.69			22.35 ± 2.62			29.00 ± 3.34		
Hyperlipemia									
N	20.19 ± 2.52	0.961	0.339	21.02 ± 2.42	1.432	0.156	28.00 ± 3.04	0.653	0.515
Y	20.73 ± 2.98			21.78 ± 2.75			28.43 ± 3.30		
Hyperglycemia									
N	20.50 ± 2.72	− 0.929	0.356	21.43 ± 2.54	− 1.053	0.295	28.23 ± 3.33	− 0.530	0.596
Y	19.80 ± 2.61			20.67 ± 2.71			27.77 ± 1.79		
Smoke									
N	20.10 ± 2.59	1.673	0.098	21.00 ± 2.43	1.837	0.069	27.86 ± 2.93	1.480	0.142
Y	21.09 ± 2.87			22.03 ± 2.77			28.87 ± 3.51		
Drink									
N	20.33 ± 2.73	1.027	0.307	21.26 ± 2.56	0.857	0.394	28.08 ± 3.14	1.146	0.255
Y	21.60 ± 2.14			22.26 ± 2.77			29.71 ± 2.91		
Inheritance									
N	20.07 ± 2.73	1.422	0.158	20.92 ± 2.60	1.773	0.079	28.13 ± 3.17	0.118	0.906
Y	20.85 ± 2.62			21.84 ± 2.45			28.21 ± 3.12		

### Correlations between the ascending aorta and sternum

The relationships between S-AAD/S-SD and cardiac phases are shown in Table S3 and Fig. 1D. The ascending aorta at the junction of the sinus tube was closest to the sternum at a cardiac phase of 45% (22.61% ± 5.69 (%)) and farthest from the sternum at 0% (27.17 ± 6.29(%)). However, the changes of S-AAD/S-SD within the cardiac cycle was not statistically significant with the* p* = 0.403.

### Reproducibility

Intraclass correlation coefficients (ICCs) of 20 cardiac R–R intervals were range from 0.934 to 0.997 for AAD(STJ), 0.956 to 0.997 for AAD, 0.992to 0.998 for DAD(Dia), 0.966 to 0.996 DAD(STJ), 0.869 to 0.997 DAD, and 0.990 to 0.996 for MPAD, respectively.

## Discussion

### Main findings

This is the first study to carry out a detailed dynamic evaluation of the aortic diameter in different planes with the reconstruction of 20 cardiac phases in a whole cardiac cycle, which could prevent 10% of the interval error and get more accurate information in the assessment of aortic disease. The diameter of the aorta changes dynamically in different phases of the cardiac cycle and matching the physiological phase of that cycle. We also found that DAD/AAD was the most stable parameter in the cardiac cycle. Moreover, the ascending aorta was the closest to the sternum at cardiac phases of 45%.

The mixed effect model simulating the real state of illness was used for statistical analysis to decompose the variation source of the measurement value with the consideration of the high correlation of the same index measurement value of the same patient in the 20 measurements to reduce false-positive results. And we found that the correlation between aorta in different planes and cardiovascular risk factors was different.

### Aortic diameter characteristics

There are seven physiological stages of the cardiac cycle. The R–R interval corresponds with Deng Wen et al.’s^16^ physiological stages of the cardiac cycle. The pressure in the aorta reaches the lowest value at the end of the isovolumic contraction. In this report, the minimum value of the diameter of the aorta in different parts mostly appears (4/5) in the phase of 0%, which corresponds to the end of the isovolumic contraction in the cardiac cycle. The minimum value of DAD appears in the phase of 95%, which still corresponds to the end of the isovolumic systolic period with low aortic pressure. The pressure of the aorta is the highest at the end of rapid ejection. However, the maximum value of the diameter of the aorta appears in the 30% and 35% phases, which approximately correspond to the end of the slow ejection period. We hold the view that the kinetic energy of the ventricular blood is high after the rapid ejection phase even if the aortic pressure starts to gradually decrease during the slow ejection phase, which could help the blood in the ventricle continue to pump into the aorta and against the pressure gradient so that the diameter of the aorta still expands.

There are many studies on MPAD/AAD, but most of them were done on the random phase of the cardiac cycle^17,18^. We found that the MPAD/AAD ranged from 0.7335 ± 0.0981 to 0.8140 ± 0.1029 rather than being constant throughout the whole cardiac cycle, which might mean that changes of MPAD/AAD in the cardiac cycle should be taken into account when evaluating pulmonary hypertension with MPAD/AAD. As for the ratio between the ascending and descending aorta, it showed that the change of DAD/AAD in a cardiac cycle was the most stable compared with the other parameters, with a range of 0.7209 ± 0.0793 to 0.7263 ± 0.0814. Therefore, we believe that DAD/AAD is most suitable for evaluating the condition of the aorta on CT thorax acquired without cardiac CT protocol.

The most drastic change of artery diameter appeared in the 10–20% phase. And the heart is in the rapid ejection phase, and the arterial pressure rises rapidly from the lowest value at the end of isovolumic contraction to the highest value at this time, which leads to the most acute change during the 10–20% R–R interval.

The aortic diameter was positively correlated with age in our study. Collagen and elastic fibers of the aorta are wavy in young people, but over time, the degradation and fracture of elastic fibers and lamellar structures^19,20^ and the accumulation and cross-linking of collagen and proteoglycans^21^, which are the final product of glycosylation^22^, lead to an increase of the artery wall hardness, a decrease of elasticity, and, eventually, the enlargement of the lumen.

In terms of the relationship between age and the aortic diameter, our results are similar to those of previous studies. In Martin et al.’s^9^ report, the average AAD expanded by 0.12 cm for every decade of life, while the corresponding value in the Aronberg et al.^23^ report was 0.1 cm. Rogers et al.^24^ found that the average AAD increases by 0.16 mm/year for women and 0.2 mm/year for men, and the average diameter of the thoracic aorta increases by 0.16 mm/year for women and 0.19 mm/year for men. The correlation between aorta in different planes and age is different in this study, it, which is believed to be caused by the different elastin content of aorta in different planes^25^. However, although some scholars also reported that the correlation between aortic diameter in different planes and BSA, gender is different^25^, which might due to difference in elastin concentration among different segments of the aorta, but the reason still needs to be explored. In addition, Mensel et al.^26^ found that smoking, glycosylated hemoglobin, and low-density lipoprotein had no significant correlation with the aortic diameter, which was consistent with this study. Hypertension generally does not involve elastic arteries except in the visceral stage. There was no statistical significance between the aorta diameters and hypertension in this study, which might be due to the fact that patients with hypertension had mild systemic arterial hypertension. These patients took drugs regularly and were in the arterial disease or even functional disorder stage.

### Correlations between the ascending aorta and the sternum

The proximity of the aorta to the sternum is a major potential cause of morbidity and mortality^27^. The stage of the aorta closest to the sternum is different from the time at which its largest diameter appears, which may indicate that the aorta is displaced during the heart beating process, and the different cardiac phases of the ascending aorta closest to the sternum in different planes might suggest that the movements of the aorta in different parts of the heart cycle are not completely synchronous. However, the study by Mao et al.^28^ showed the aorta is closest to the sternum at the 35% phase and the farthest from the sternum at the 95% phase. This may be because the Mao study directly assessed the S-AAD while this study was evaluated using ratios, thus excluding the effects of aortic displacement caused by respiratory movements.

There are some limitations to this study. The most obvious limitation of this study was the radiation dose generated by using CT, although we had radiation protection for our patients, and the radiation dose was at a safe level. Moreover, it was a cross-sectional study, and only patients with clear image quality were evaluated. Furthermore, measurement of the aortic diameters was manual in this study. However, the average of the two measurements was used for statistical analysis to reduce measurement bias, and manual measurement is a simple and convenient method for clinical routine measurement.

In conclusion, the diameter of the aorta changes with the cardiac cycle, and the aorta maximum value appears at the cardiac phases of 30% and 35%. At those cardiac phases, reconstruction and measurement, which can be performed using cardiac CT, could result in a more accurate aortic diameter in aortic disease assessment. Results also showed that the ratio of the DAD to AAD in the plane of pulmonary artery bifurcation was the most stable parameter in a cardiac cycle.

## Materials and methods

In accordance with the Declaration of Helsinki (2013 EDITION), the institutional review board in our hospital approved our study. Because of the retrospective nature of the study, we were not required to get signed patient informed consent.

### Patient selection

The study included 300 consecutive subjects who underwent cardiac CT for suspected coronary artery disease between June 2019 and November 2019. Patients with negative results for coronary artery examination and image quality scores ≥ 2 were enrolled. Enrollment decisions were not affected by body mass index, and patients with mild systemic arterial hypertension who took regular medication and those with other histories of traditional cardiovascular risk factors were included. Exclusion criteria included patients with severe arrhythmia, pacemakers, implantable devices, previous thoracic endografts, surgical procedures of the aorta, known aortopathies, or heart disease. Patients with structural or functional abnormalities of the heart and aorta detected by echocardiography were also excluded. Ninety-seven patients (58 men and 39 women) were ultimately recruited for this study.

The following is an explanation of the image quality scores of the thoracic aorta:

- 0: The motion artifact is serious, the lumen and wall of the artery are fuzzy, the boundary of the vessel wall cannot be determined, and measurement is impossible.
- 1: There is no motion artifact, but the wall of the artery (mainly thoracic aorta) is calcified.
- 2: Four cardiac phases at most have large motion artifacts, the edge of the arterial wall is clear and can be measured, and the other cardiac phase image scores are ≤ 1.
- 3: There is a slight motion artifact, but the edge of the artery wall is basically clear and does not affect the measurement.
- 4: The artery is clearly developed, the artery wall is clear, there is no motion artifact, and there is no calcified plaque.

### CT protocol

All patients were scanned from the bifurcation of the pulmonary artery to the level of the diaphragm with a thickness of 0.5 cm using a 256-slice scanner (Philips Brilliance iCT; Philips Medical Systems, Cleveland, OH, USA) with retrospective ECG-gating. Acquisitions were obtained during a single breath-hold after the injection of the contrast agent iodixanol (1.0–1.2 ml/kg; General Electric Pharmaceutical Co. Ltd., Shanghai, China) by contrast agent monitoring technology, which set the descending aorta as the trigger point (the threshold value was set as 250 hu). We used 120 kv for patients with body weight ≥ 75 kg, 100 kV for patients with body weight < 7 kg, 1200 mas/slice for patients with body mass index (BMI) ≥ 24, and 900 mas/slice for patients with BMI < 24. Multi-phase reconstructions were done from 0 to 95% in increments of 5% on a 256 slice spiral CT scanning workstation, and the reconstruction vision of 20 cardiac cycles was extended to the whole thorax.

### Imaging analysis

**Figure 2:**
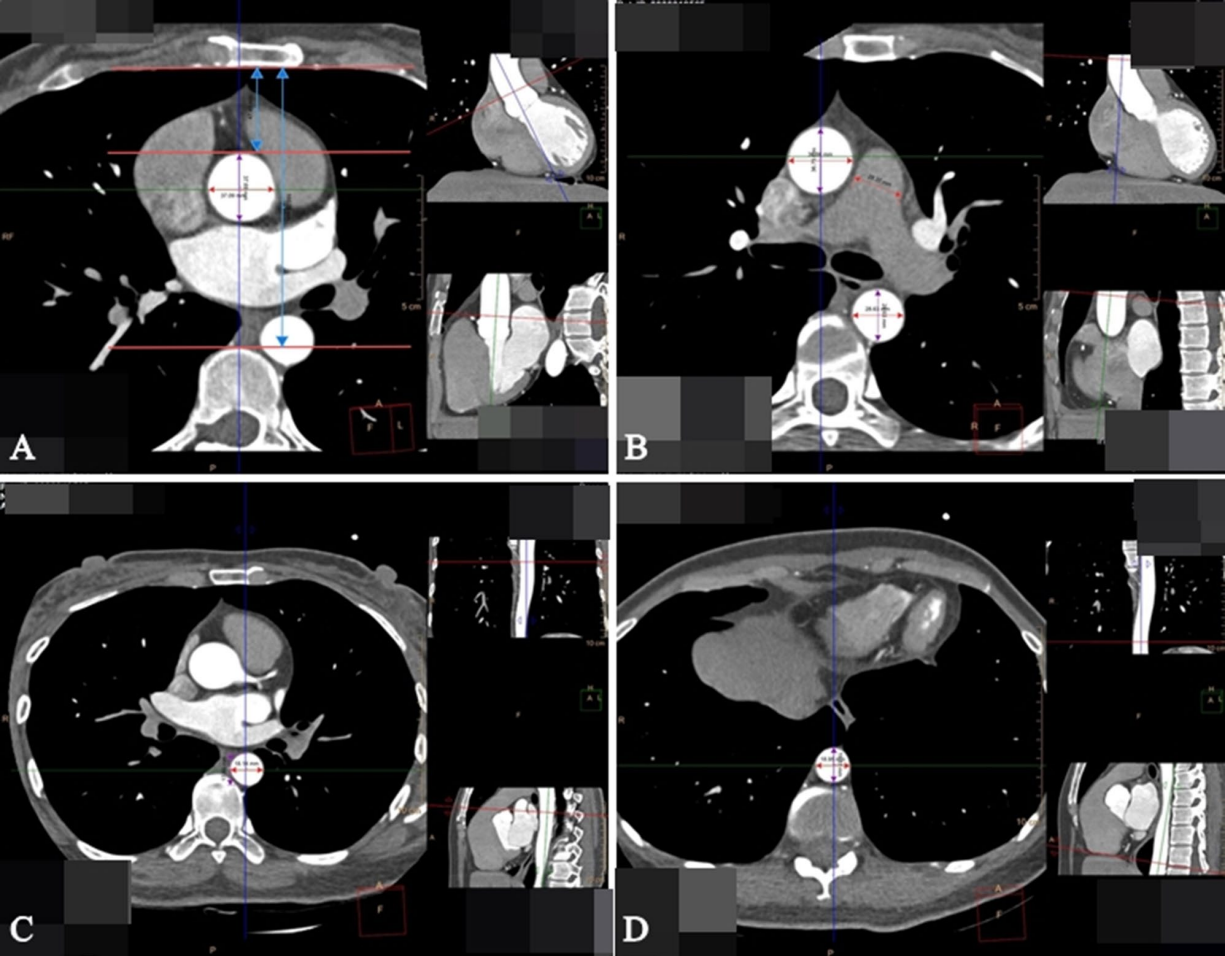
Axial contrast enhanced CT thorax with cardiac gated CT protocol were performed and representative slices were include at the levels of (**A**) The plane of sinus junction—the transverse and anteroposterior diameter of the ascending aorta, the distance from sternum to ascending aorta and to spinal distances. (**B**) The plane of pulmonary bifurcation—the transverse and anteroposterior diameter of the ascending aorta, descending aorta, and the diameter of the main pulmonary artery. (**C**) The plane of sinus junction—the transverse and anteroposterior diameter of descending aorta. (**D**) The plane of the diaphragm—the transverse and anteroposterior diameter of descending aorta.

All images were transferred to a post-processing workstation (Philips Intelli Space Portal system) and loaded into the cardiac viewer application, which allowed three perpendicular axes to be adjusted simultaneously to obtain perpendicular imaging planes for measurement. The diameters of two orthogonal dimensions of the aorta in different planes were measured and averaged, including the diameter of ascending aorta (AAD) in the plane of pulmonary bifurcation, the diameter of the main pulmonary artery (MPAD), the diameter of the descending aorta (DAD) in the plane of pulmonary bifurcation, the AAD in the plane of the sinus junction (AAD [STJ]), the DAD in the plane of the sinus junction (DAD [STJ]), and DAD in the plane of the diaphragm (DAD [Dia]). In the plane of the ascending aorta, the antero-posterior distance from the posterior border of the sternum to the anterior edge of the spine (S-SD) and the vertical distance from the posterior border of the sternum to the anterior edge of the ascending aorta (S-AAD) in the plane of pulmonary artery bifurcation were measured separately (Fig. 2).

Two experienced radiologists (both with 3 years of experience in the interpretation of cardiac CT images), who were blind to clinical data, independently reviewed the reconstructed CT images on a dedicated post-processing workstation. The average value measured by the two readers was taken. The change rate (CR) of different parameters in every 5% R–R interval was calculated: CR = |posterior cardiac coherence value − anterior cardiac coherence value|/anterior cardiac coherence value × 100% (example: AAD_CR (0%~5%)_ = |AAD_5%_ − AAD_0%_|/AAD_0%_ × 100%).

### Statistical analysis

Statistical software R (Version 3.6.1, R Core Team, 2019) was used for statistical description and inference. ICCs were used to evaluate the reproducibility of intra-observer variability. Qualitative data were expressed by frequency and percentage, and the quantitative data were expressed by mean ± standard deviation. A general linear mixed model was used to explore the differences in the diameter of the aorta in different cardiac phases and to analyze the correlation between artery diameter and traditional risk factors. p < 0.05 was considered statistically significant.

### Ethical approval

The Institutional Review Board of the Affiliated Hospital of Southwest Medical University has approved the waived of the informed consent.

## Supplementary Information

Supplementary Information 1.

Supplementary Information 2.

Supplementary Information 3.
