# Supplementary material for: Assessment of thoracic aorta in different cardiac phases in patients with non-aorta diseases using cardiac CT
Source: Sci Rep. 2021 Jul 26;11:15209. doi: 10.1038/s41598-021-94677-5 (PMC8313572; doi:10.1038/s41598-021-94677-5)
Supplement: Supplementary file 1 — Supplementary Information 1. [file 41598_2021_94677_MOESM1_ESM.docx]

**Data supplements-Additional tables**

**Title page**

**Title: Assessment of Thoracic Aorta in Different Cardiac Phases in Patients with Non-aorta Diseases using Cardiac CT**

Xue Zheng**^1#^**, MD; Yu-jiao Deng^1^**^#^**, MD; Fu-Gang Han^1^,MD; Jin-Rong Zhou^1^, MD; Li Luo^1^,MD;Jing Chen ^1^*,MD. Ph.D

1. Department of Radiology, The Affiliated Hospital of Southwest Medical University,25# Tai Ping Street, Luzhou, Sichuan 646099, China.

**E-mail address of all authors:**

Xue Zheng, [1078490504@qq.com](mailto:1078490504@qq.com),

Yu-jiao Deng, [1278571224@qq.com](mailto:1278571224@qq.com)

Fu-Gang Han, [8311hfg@163.com](mailto:8311hfg@163.com)

Jin-Rong Zhou, [zjr61777@163.com](mailto:zjr61777@163.com)

Li Luo, [drflora94@163.com](mailto:drflora94@163.com)

Jing Chen, [yzqdcj@126.com;](mailto:yzqdcj@126.com;)

**Table S1 Relationship between aortic diameter and cardiac cycle**

**Diameter**

**(mm)**

| **Cardiac**  **Cycle (%)** | **AAD(STJ)** | **AAD** | **DAD(Dia)** | **DAD (STJ)** | **DAD** |
| --- | --- | --- | --- | --- | --- |
| 0 | 27.44±3.16 | 30.83±3.85 | 19.80±2.71 | 20.75±2.61 | 22.26±2.93 |
| 5 | 27.58±3.22 | 30.93±3.80 | 19.84±2.73 | 20.84±2.61 | 22.29±2.90 |
| 10 | 27.88±3.13 | 31.25±3.69 | 20.00±2.74 | 21.02±2.60 | 22.47±2.92 |
| 15 | 28.28±3.08 | 31.72±3.51 | 20.34±2.71 | 21.29±2.62 | 22.77±2.90 |
| 20 | 28.57±3.13 | 32.04±3.40 | 20.73±2.73 | 21.61±2.55 | 23.11±2.92 |
| 25 | 28.79±3.10 | 32.33±3.40 | 20.95±2.68 | 21.76±2.56 | 23.31±2.88 |
| 30 | 28.86±3.01 | 32.47±3.35 | 21.05±2.66 | 21.84±2.53 | 23.41±2.87 |
| 35 | 28.81±3.10 | 32.51±3.35 | 21.09±2.66 | 21.85±2.58 | 23.46±2.88 |
| 40 | 28.72±3.05 | 32.38±3.36 | 20.93±2.65 | 21.75±2.55 | 23.36±2.87 |
| 45 | 28.59±3.08 | 32.27±3.41 | 20.84±2.66 | 21.64±2.55 | 23.21±2.84 |
| 50 | 28.52±3.08 | 32.04±3.48 | 20.69±2.69 | 21.51±2.54 | 23.10±2.85 |
| 55 | 28.38±3.12 | 31.94±3.49 | 20.61±2.67 | 21.47±2.56 | 23.01±2.83 |
| 60 | 28.29±3.11 | 31.79±3.57 | 20.48±2.70 | 21.39±2.5 | 22.93±2.82 |
| 65 | 28.15±3.14 | 31.65±3.56 | 20.37±2.68 | 21.34±2.52 | 22.79±2.87 |
| 70 | 28.00±3.14 | 31.50±3.60 | 20.29±2.69 | 21.23±2.52 | 22.68±2.87 |
| 75 | 27.92±3.14 | 31.40±3.64 | 20.19±2.67 | 21.16±2.56 | 22.58±2.87 |
| 80 | 27.79±3.11 | 31.27±3.69 | 20.06±2.71 | 21.06±2.55 | 22.48±2.87 |
| 85 | 27.71±3.19 | 31.11±3.69 | 20.00±2.72 | 20.98±2.59 | 22.39±2.82 |
| 90 | 27.52±3.19 | 30.95±3.74 | 19.87±2.71 | 20.88±2.55 | 22.29±2.84 |
| 95 | 27.45±3.22 | 30.86±3.77 | 19.79±2.69 | 20.81±2.64 | 22.24±2.88 |
| **t *** | -10.552 | -8.869 | -9.278 | -8.024 | -9.855 |
| **P** | <0.001 | <0.001 | <0.001 | <0.001 | <0.001 |

**Note: *: t value is the fixed effect coefficient score test statistic of variable "cardiac cycle" in general linear mixed model, that is, it measures the difference of the effect index measured when the cardiac cycle is different in vivo.**

**Table S2 Relationship between the rate of change of each aortic diameter and the cardiac cycle**

| **CR (†)**  **Cardiac**  **Cycle (%)** | **AAD(STJ)** | **AAD** | **DAD(Dia)** | **DAD (STJ)** | **DAD** |
| --- | --- | --- | --- | --- | --- |
| 0-5 | 1.13±0.95 | 0.88±0.91 | 0.78±0.71 | 1.07±0.97 | 0.82±0.64 |
| 5-10 | 1.62±1.36 | 1.28±1.20 | 0.99±0.73 | 1.29±1.34 | 1.13±0.89 |
| 10-15 | 1.67±1.65 | 1.93±1.68 | 1.85±1.51 | 1.64±1.47 | 1.48±1.29 |
| 15-20 | 1.37±1.17 | 1.34±1.28 | 2.02±1.48 | 1.73±1.37 | 1.60±1.17 |
| 20-25 | 1.22±1.12 | 1.14±1.01 | 1.36±1.18 | 1.00±0.97 | 1.20±1.02 |
| 25-30 | 0.98±0.91 | 0.86±0.97 | 0.98±0.86 | 0.93±0.85 | 0.73±0.62 |
| 30-35 | 1.00±1.09 | 0.74±0.97 | 0.78±0.56 | 0.92±0.88 | 0.67±0.59 |
| 35-40 | 1.09±1.05 | 0.69±0.62 | 0.92±0.76 | 0.89±0.70 | 0.84±0.69 |
| 40-45 | 1.04±1.02 | 0.67±0.58 | 0.88±0.66 | 0.81±0.67 | 0.82±0.72 |
| 45-50 | 1.17±1.08 | 0.97±1.02 | 1.05±0.73 | 0.83±0.71 | 0.82±0.68 |
| 50-55 | 1.06±0.90 | 0.78±0.58 | 0.86±0.73 | 0.87±0.79 | 0.70±0.64 |
| 55-60 | 1.09±1.22 | 0.78±0.72 | 0.95±0.68 | 0.88±0.81 | 0.70±0.62 |
| 60-65 | 0.94±0.95 | 0.71±0.54 | 0.80±0.62 | 0.79±0.74 | 0.86±0.71 |
| 65-70 | 0.98±0.74 | 0.73±0.66 | 0.72±0.66 | 0.84±0.85 | 0.88±0.69 |
| 70-75 | 0.80±0.66 | 0.57±0.43 | 0.81±0.76 | 0.91±0.78 | 0.79±0.70 |
| 75-80 | 0.86±0.64 | 0.61±0.55 | 0.92±0.72 | 0.90±0.85 | 0.80±0.70 |
| 80-85 | 0.80±0.60 | 0.68±0.48 | 0.83±0.56 | 0.86±0.67 | 0.83±0.69 |
| 85-90 | 1.12±0.90 | 0.73±0.62 | 0.89±0.75 | 0.92±0.82 | 0.84±0.62 |
| 90-95 | 1.05±0.94 | 0.70±0.57 | 0.94±0.66 | 0.93±0.86 | 0.79±0.82 |
| 95-0 | 1.14±1.00 | 0.75±0.54 | 0.96±0.91 | 1.20±1.10 | 0.97±0.78 |
| **t *** | -5.527 | -8.937 | -7.276 | -5.292 | -6.129 |
| **P** | <0.001 | <0.001 | <0.001 | <0.001 | <0.001 |

**Note: *: t value is the fixed effect coefficient score test statistic of variable "cardiac cycle" in general linear mixed model, that is, it measures the difference of the effect index measured when the cardiac cycle is different in vivo. †: the unit scale is one ten thousandth, which shows the variation degree of each index more accurately. CR: Chang Rate.**

**Table S3 Relationship between aortic ratio and sternal distance ratio and cardiac cycle**

| **Ratio**  **(%)**  **Cardiac**  **Cycle (%)** | **MPAD/AAD** | **DAD/AAD(STJ)** | **DAD/AAD** | **S-AAD/**  **S-SD(STJ)** | **S-AAD/S-SD** |
| --- | --- | --- | --- | --- | --- |
| 0 | 77.00±10.36 | 75.98±8.16 | 72.63±8.14 | 35.92±5.11 | 27.17±6.29 |
| 5 | 78.49±10.22 | 75.93±8.14 | 72.48±8.11 | 35.75±5.11 | 27.21±6.22 |
| 10 | 79.59±10.58 | 75.74±8.18 | 72.29±8.01 | 35.16±5.24 | 26.42±6.11 |
| 15 | 80.63±10.37 | 75.60±8.14 | 72.09±7.93 | 33.86±5.16 | 25.39±5.81 |
| 20 | 81.40±10.29 | 75.98±7.97 | 72.41±7.89 | 32.61±5.01 | 24.48±5.89 |
| 25 | 80.71±10.20 | 75.93±8.21 | 72.41±7.94 | 31.58±4.88 | 23.76±5.77 |
| 30 | 79.51±9.63 | 75.98±7.86 | 72.41±7.83 | 30.72±4.96 | 23.15±5.68 |
| 35 | 78.44±9.83 | 76.17±8.07 | 72.44±7.75 | 30.08±4.92 | 22.65±5.62 |
| 40 | 77.44±9.79 | 76.07±8.03 | 72.44±7.89 | 29.89±4.83 | 22.61±5.69 |
| 45 | 76.97±10.03 | 76.05±8.13 | 72.24±7.76 | 29.79±4.84 | 22.67±5.68 |
| 50 | 77.28±10.02 | 75.76±8.10 | 72.44±7.90 | 30.37±4.94 | 23.13±5.85 |
| 55 | 77.27±9.91 | 76.01±8.02 | 72.39±7.83 | 31.21±5.18 | 23.71±5.97 |
| 60 | 76.39±9.73 | 75.98±7.88 | 72.51±7.83 | 32.36±5.13 | 24.34±6.07 |
| 65 | 75.53±10.23 | 76.16±7.85 | 72.38±7.98 | 32.90±4.94 | 24.65±6.06 |
| 70 | 74.61±9.85 | 76.18±7.89 | 72.40±7.95 | 33.04±4.93 | 24.99±6.05 |
| 75 | 73.88±9.93 | 76.11±7.88 | 72.30±8.03 | 33.12±4.90 | 25.08±5.96 |
| 80 | 73.35±9.81 | 76.11±7.85 | 72.29±8.01 | 33.20±4.92 | 25.27±6.02 |
| 85 | 73.62±10.50 | 76.07±8.01 | 72.39±8.06 | 33.41±4.87 | 25.45±6.01 |
| 90 | 74.44±10.37 | 76.23±7.99 | 72.46±8.08 | 34.09±5.05 | 25.92±6.10 |
| 95 | 75.59±10.39 | 76.20±8.39 | 72.50±8.07 | 35.20±5.01 | 26.65±6.18 |
| **t *** | **-17.661** | **2.217** | 0.023 | **-2.338** | -0.838 |
| **P** | **<0.001** | **0.029** | 0.982 | **0.020** | 0.403 |

**Note: *: t value is the fixed effect coefficient score test statistic of variable "cardiac cycle" in general linear mixed model, that is, it measures the difference of the effect index measured when the cardiac cycle is different in vivo.**

**Table S4 Relationship between the rate of change of aortic ratio and cardiac cycle**

| **Cardiac**  **Cycle (%)**  **CR (†)** | **MPAD/AAD** | **DAD/AAD(STJ)** | **DAD/AAD** | **S-AAD/**  **S-SD(STJ)** | **S-AAD/S-SD** |
| --- | --- | --- | --- | --- | --- |
| 0 | 3.24±3.17 | 1.53±1.14 | 1.19±1.12 | 1.88±2.16 | 1.38±1.25 |
| 5 | 2.98±2.86 | 1.56±1.57 | 1.39±1.13 | 3.49±3.39 | 2.57±2.35 |
| 10 | 3.27±3.24 | 1.70±1.61 | 1.46±1.30 | 4.54±3.98 | 4.08±3.57 |
| 15 | 3.07±2.88 | 1.67±1.27 | 1.41±1.23 | 4.17±4.48 | 4.14±4.17 |
| 20 | 2.11±1.79 | 1.38±1.01 | 1.12±0.85 | 3.71±3.38 | 3.83±3.67 |
| 25 | 2.23±1.92 | 1.37±1.16 | 1.06±1.17 | 3.48±2.90 | 3.77±3.56 |
| 30 | 2.29±2.10 | 1.37±1.32 | 1.03±1.04 | 2.78±2.89 | 2.80±2.55 |
| 35 | 2.29±1.92 | 1.18±1.07 | 1.02±0.79 | 1.84±1.93 | 1.83±1.63 |
| 40 | 1.86±1.49 | 1.22±1.12 | 1.02±0.82 | 1.95±1.70 | 1.84±1.61 |
| 45 | 2.31±2.26 | 1.24±1.01 | 1.02±0.88 | 3.11±3.72 | 3.44±4.42 |
| 50 | 2.36±2.19 | 1.32±1.03 | 0.88±0.77 | 3.26±3.02 | 3.62±4.10 |
| 55 | 2.62±2.50 | 1.40±1.43 | 0.93±0.81 | 3.44±3.53 | 4.83±4.55 |
| 60 | 2.57±2.56 | 1.25±1.17 | 0.87±0.74 | 2.22±2.91 | 2.79±3.31 |
| 65 | 2.21±2.21 | 1.19±0.96 | 0.85±0.71 | 2.34±2.40 | 1.78±1.69 |
| 70 | 2.31±1.87 | 1.13±1.05 | 0.85±0.79 | 1.51±1.61 | 1.22±1.26 |
| 75 | 1.74±1.97 | 1.06±1.08 | 0.84±0.80 | 1.51±1.55 | 1.26±1.40 |
| 80 | 1.92±1.97 | 0.95±0.76 | 0.87±0.75 | 1.75±1.83 | 1.60±1.81 |
| 85 | 2.58±2.75 | 1.30±1.00 | 0.90±0.74 | 2.57±2.88 | 2.68±2.80 |
| 90 | 2.67±2.09 | 1.47±1.34 | 0.98±0.87 | 3.47±3.12 | 4.14±3.90 |
| 95 | 3.42±3.48 | 1.87±1.64 | 1.22±0.94 | 2.93±2.97 | 2.76±2.75 |
| **t *** | -1.956 | -2.069 | -4.778 | -5.259 | -2.727 |
| **P值** | 0.053 | 0.041 | <0.001 | <0.001 | 0.007 |

**Note: *: t value is the fixed effect coefficient score test statistic of variable "cardiac cycle" in general linear mixed model, that is, it measures the difference of the effect index measured when the cardiac cycle is different in vivo. †: the unit scale is one ten thousandth, which shows the variation degree of each index more accurately. CR: Change Rate**

**Table S5 Multi-factor general linear mixed effects model of AAD correlation factors**

| **Variable** | **B** | **SE** | **df** | **t** | **P** |
| --- | --- | --- | --- | --- | --- |
| **Fixed effect** |  |  |  |  |  |
| Age | 0.156 | 0.032 | 94 | 4.922 | <0.001 |
| Hypertension | 0.580 | 0.722 | 94 | 0.803 | 0.424 |
| Cardiac cycle | -0.025 | 0.003 | 1842 | -9.106 | <0.001 |
| Intercept | 23.417 | 1.653 | 94 | 14.163 | <0.001 |
| **Random effects** |  |  |  |  |  |
| Intercept-individual | 9.601 | 3.099 |  |  |  |
| Intercept-Cardiac cycle | 0.471 | 0.686 |  |  |  |

**Table S6 Multi-factor general linear mixed effects model of DAD correlation factors**

| **Variable** | **B** | **SE** | **df** | **t** | **P** |
| --- | --- | --- | --- | --- | --- |
| **Fixed effect** |  |  |  |  |  |
| Age | 0.130 | 0.022 | 91 | 5.798 | **<0.001** |
| BSA | 7.743 | 2.271 | 91 | 3.410 | **<0.001** |
| BMI | 0.031 | 0.099 | 91 | 0.316 | 0.753 |
| Gender | 0.734 | 0.613 | 91 | 1.197 | 0.235 |
| Hypertension | 0.683 | 0.492 | 91 | 1.389 | 0.168 |
| Cardiac cycle | -0.019 | 0.002 | 1842 | -10.010 | <0.001 |
| Intercept | 1.421 | 2.896 | 91 | 0.491 | 0.625 |
| **Random effects** |  |  |  |  |  |
| Intercept-individual | 4.223 | 2.055 |  |  |  |
| Intercept-Cardiac cycle | 0.224 | 0.474 |  |  |  |

**Table S7 Multi-factor general linear mixed effects model of DAD(Dia) correlation factors**

| **Variable** | **B** | **SE** | **df** | **t** | **P** |
| --- | --- | --- | --- | --- | --- |
| **Fixed effect** |  |  |  |  |  |
| Age | 0.138 | 0.020 | 91 | 6.908 | <0.001 |
| BSA | 6.515 | 2.025 | 91 | 3.218 | 0.018 |
| BMI | 0.020 | 0.088 | 91 | 0.226 | 0.822 |
| Gender | 1.232 | 0.547 | 91 | 2.253 | 0.027 |
| Hypertension | 0.401 | 0.439 | 91 | 0.915 | 0.362 |
| Cardiac cycle | -0.018 | 0.002 | 91 | -9.279 | <0.001 |
| Intercept | 0.742 | 2.582 | 1842 | 0.287 | 0.775 |
| **Random effects** |  |  |  |  |  |
| Intercept-individual | 3.353 | 1.831 |  |  |  |
| Intercept-Cardiac cycle | 0.242 | 0.492 |  |  |  |

**Table S8 Multi-factor general linear mixed effects model of DAD(STJ) correlation factors**

| **Variable** | **B** | **SE** | **df** | **t** | **P** |
| --- | --- | --- | --- | --- | --- |
| **Fixed effect** |  |  |  |  |  |
| Age | 0.111 | 0.020 | 91 | 5.595 | <0.001 |
| BSA | 5.994 | 2.008 | 91 | 2.985 | 0.004 |
| BMI | 0.051 | 0.087 | 91 | 0.586 | 0.560 |
| Gender | 1.120 | 0.542 | 91 | 2.061 | 0.042 |
| Hypertension | 0.540 | 0.435 | 91 | 1.242 | 0.217 |
| Cardiac cycle | -0.014 | 0.002 | 91 | -8.061 | <0.001 |
| Intercept | 3.218 | 2.561 | 1842 | 1.256 | 0.212 |
| **Random effects** |  |  |  |  |  |
| Intercept-individual | 3.303 | 1.817 |  |  |  |
| Intercept-Cardiac cycle | 0.190 | 0.436 |  |  |  |

**Table S9 Multi-factor general linear mixed effects model of AAD(STJ) correlation factors**

| **Variable** | **B** | **SE** | **df** | **t** | **P** |
| --- | --- | --- | --- | --- | --- |
| **Fixed effect** |  |  |  |  |  |
| Age | 0.111 | 0.027 | 93 | 4.081 | <0.001 |
| BSA | 4.022 | 1.847 | 93 | 2.178 | 0.032 |
| Gender | 1.548 | 0.693 | 93 | 2.233 | 0.028 |
| Cardiac cycle | -0.025 | 0.002 | 93 | -10.649 | <0.001 |
| Intercept | 14.665 | 3.670 | 1842 | 3.996 | 0.001 |
| **Random effects** |  |  |  |  |  |
| Intercept-individual | 7.344 | 2.710 |  |  |  |
| Intercept-Cardiac cycle | 0.348 | 0.590 |  |  |  |
