# Supplementary figures and images for: Assessment of thoracic aorta in different cardiac phases in patients with non-aorta diseases using cardiac CT
Source: Sci Rep. 2021 Jul 26;11:15209. doi: 10.1038/s41598-021-94677-5 (PMC8313572; doi:10.1038/s41598-021-94677-5)

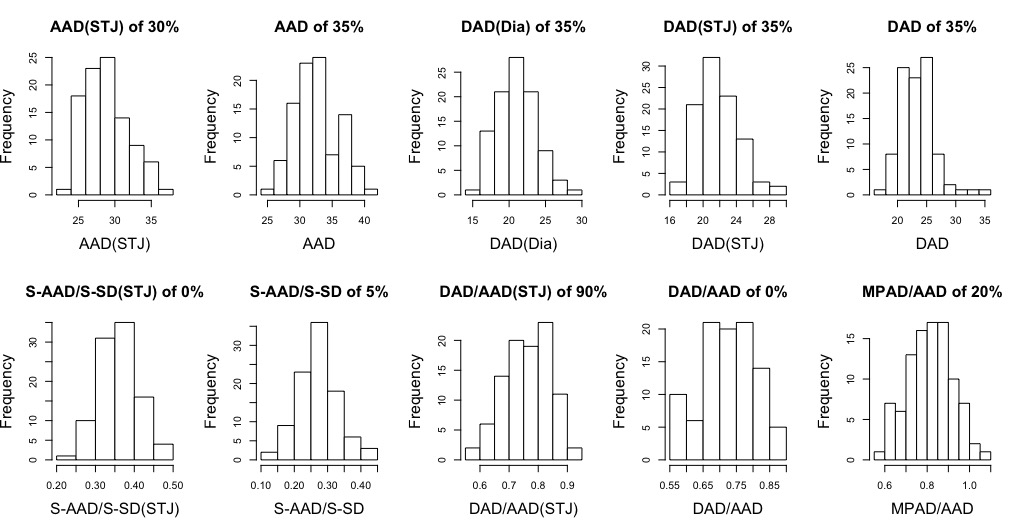

Supplement: Supplementary file 3 — Supplementary Information 3. [file 41598_2021_94677_MOESM3_ESM.tif]
